# Human umbilical cord-derived mesenchymal stem cells ameliorate insulin resistance via PTEN-mediated crosstalk between the PI3K/Akt and Erk/MAPKs signaling pathways in the skeletal muscles of db/db mice

**DOI:** 10.1186/s13287-020-01865-7

**Published:** 2020-09-16

**Authors:** Guang Chen, Xiao-yan Fan, Xiao-peng Zheng, Yue-lei Jin, Ying Liu, Shuang-chun Liu

**Affiliations:** 1 Department of Basic Medical Sciences, Taizhou University Hospital, Taizhou University, No 1139 Shifu Road, Jiaojiang District, Taizhou, 318000 China; 2 Department of Basic Medical Sciences, Jiamusi University, No 148 Xuefu road, Xiangyang District, Jiamusi, 154007 China; 3 Jilin Tuhua Bioengineering Company Limited, Shiling Town, Tiedong District, Siping, Jilin 136000 China; 4 Municipal Hospital Affiliated to Medical School of Taizhou University, No 381, Zhongshan east road, Jiaojiang district, Taizhou, 318000 China

**Keywords:** HUC-MSCs, Insulin resistant, PTEN, crosstalk, PI3K/Akt, Erk/MAPK, Db/db mice

## Abstract

**Background:**

Globally, 1 in 11 adults have diabetes mellitus, and 90% of the cases are type 2 diabetes mellitus. Insulin resistance is a central defect in type 2 diabetes mellitus, and although multiple drugs have been developed to ameliorate insulin resistance, the limitations and accompanying side effects cannot be ignored. Thus, more effective methods are required to improve insulin resistance.

**Methods:**

In the current study, db/m and db/db mice were injected with human umbilical cord-derived mesenchymal stem cells (HUC-MSCs) via tail vein injection, intraperitoneal injection, and skeletal muscle injection. Body weight, fasting blood glucose, and the survival rates were monitored. Furthermore, the anti-insulin resistance effects and potential mechanisms of transplanted HUC-MSCs were investigated in db/db mice in vivo.

**Results:**

The results showed that HUC-MSC transplantation by skeletal muscle injection was safer compared with tail vein injection and intraperitoneal injection, and the survival rate reached 100% in the skeletal muscle injection transplanted mice. HUC-MSCs can stabilize localization and differentiation in skeletal muscle tissue and significantly ameliorate insulin resistance. Potential regulatory mechanisms are associated with downregulation of inflammation, regulating the balance between PI3K/Akt and ERK/MAPK signaling pathway via PTEN, but was not associated with the IGF-1/IGF-1R signaling pathway.

**Conclusions:**

These results suggest HUC-MSC transplantation may be a novel therapeutic direction to prevent insulin resistance and increase insulin sensitivity, and skeletal muscle injection was the safest and most effective way.

## Introduction

Diabetes mellitus is a life-long metabolic disease with high morbidity and mortality rates, and DM reduces the patients’ quality of life due to acute and chronic complications [1, 2]. Type 2 diabetes mellitus (T2DM) accounts for 90–95% of diabetes mellitus cases, affecting ~ 380 million individuals worldwide [3].

Insulin resistance (IR) and relative insulin deficiency are the pathological consequence in T2DM, and these conditions are concurrent and interacting conditions. Insulin has two different functions: firstly, hepatic glucose output or glucose uptake and utilization can be inhibited or impaired by insulin. As a result, insulin levels continued to rise chronically; secondly, it begins to fail for the pancreatic β-cells regulating a hyperinsulinemic state [4, 5]. Compared with nondiabetic individuals, we rarely found the hypoinsulinemic condition in T2DM patients [4]. Additionally, a landmark study found that a degenerative process, which is a relatively small decrease in β-cell mass, affected levels of fasting blood glucose [6]. Dysregulation of the insulin signaling cascade results in the development of IR [7], which has important consequences on the regulation of glucose and lipid metabolism. There are two major pathways of insulin receptor signal transduction: the insulin receptor substrate (IRS)-PI3K-Akt (also known as PKB) pathway and the growth factor receptor-bound protein 2 (Grb2)-son of sevenless homologue 1 (SOS)-Ras-MAPK pathway (also known as the ERK pathway) [8, 9]. As a central node, Akt regulates the biological effects of insulin. Insulin can regulate cell proliferation and differentiation by Grb2-SOS-Ras pathway inducing the activation of the MAPK pathway. In addition, insulin can also activate negative regulators of insulin signal transduction to inhibit the signal pathway in the critical nodes of insulin receptor-IRS/Akt. Thus, controlling insulin signal transduction tightly is an important step to avoid severe disorder of metabolism and proliferation. Dysregulation of negative regulators in insulin signal transduction can induce the development of IR, correlated with chronic hyperactivation. These negative regulators include PTEN. PTEN can inhibit the activation of PI3K/v-Akt murine thymoma viral oncogene homologue (Akt) pathway, resulting in reduced insulin sensitivity [10]. Moreover, relative studies reported that level of PTEN increased in type 1 diabetic mice induced by the aortas of streptozotocin [11] and skeletal muscles of T2DM mice [12, 13].

**Fig. 1:**
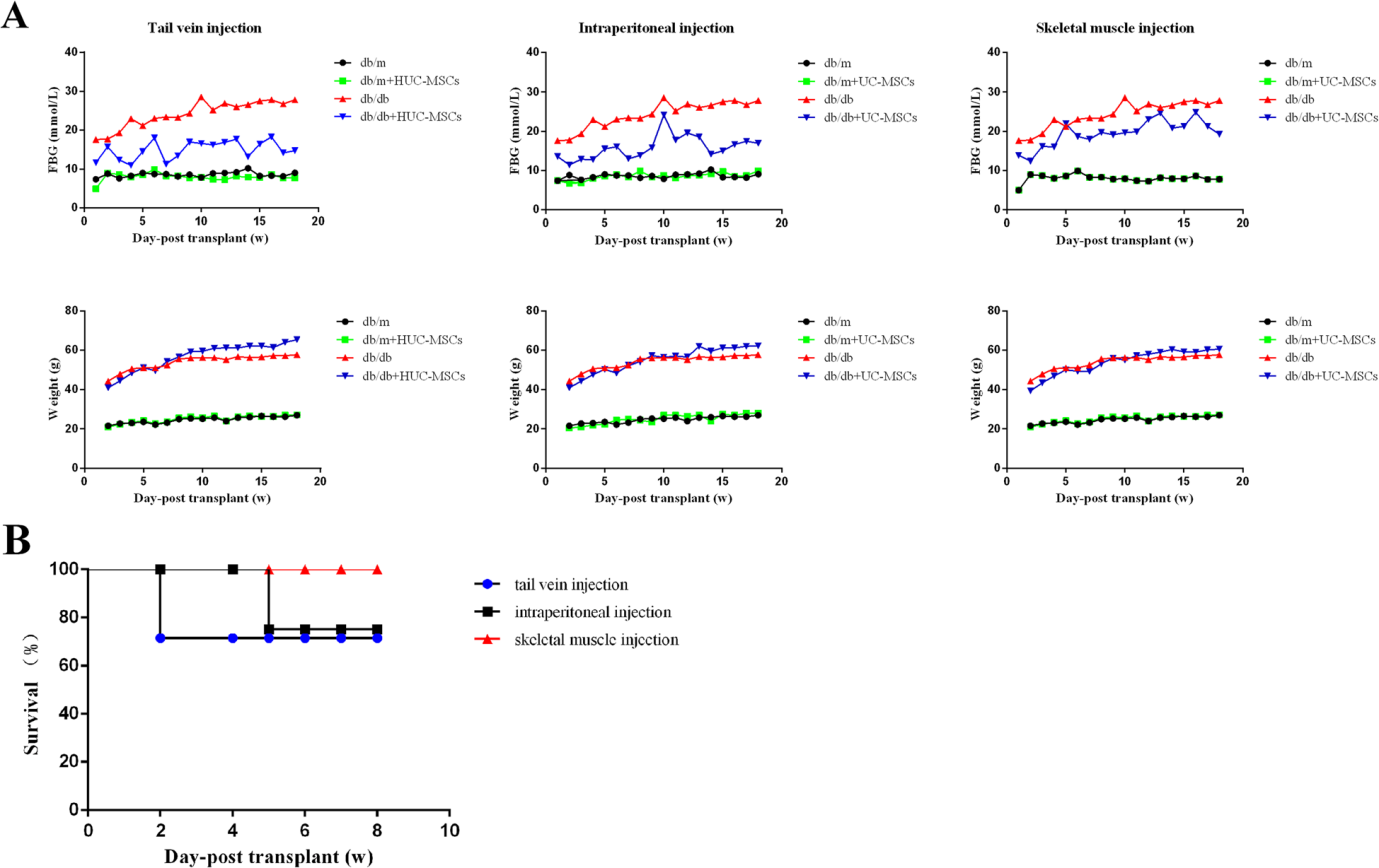
Therapeutic effects of HUC-MSC administration route in db/db mice. **a** Body weight and FBG in experimental mice with HUC-MSC transplantation by IV, IP, and IM. **b** Survival rates of mice administered with HUH-MSCs via the different routes. Data are representative of three repeats. IV, tail vain injection; IP, intraperitoneal injection; IM, skeletal muscle injection. HUC-MSC, human umbilical cord-mesenchymal stem cells; FBG, fasting blood glucose; IV, intravenous; IP, intra-peritoneal; IM, intramuscular

With the development of research technologies, epigenetics studies showed that stem cells are pluripotent, and this is achieved via cellular reprogramming techniques. Thus, alternative therapeutic approaches allowed for new advances and improved understanding of stem cell therapy [14]. At present, human umbilical cord-derived mesenchymal stem cells (HUC-MSCs) are widely used in clinical treatments as they are a rich source of self-replicating cells with a high degree of differentiation potential and extremely low immunogenicity and immunoreactivity [15]. Several studies have shown that treatment with HUC-MSCs showed impressive therapeutic effects in diabetes [16, 17] (Fig. 1). HUC-MSCs can control blood sugar levels effectively via restoring islet function and ameliorating IR.

In the present study, the treatment and anti-IR effects of HUC-MSCs were further investigated in db/db mice. The multiple targeted mechanisms of HUC-MSCs in vivo were also investigated and may serve as potential treatments for IR in T2DM.

## Materials and methods

### Reagents

Mouse insulin (EMD Millipore), TNF-α and IL-6 (Abcam) ELISA kits, anti-DNA (cat. no. AC30-10) monoclonal antibody (Novus Biologicals, Ltd.), D-Luciferin, sodium salt D-Fluorescein sodium salt (cat. no. 40901ES01; Shanghai Yeasen), LUC-labeled lentiviral particles (cat. No. GM-0220PC, Shanghai; Genomeditech), RIPA lysis buffer, nitrocellulose membranes, and an SABC (rabbit IgG)-POD kit were obtained from Beijing Solarbio Science & Technology Co., Ltd. A blood glucose meter and test strips were acquired from ACCU-CHEK (Accu-ChekSoftclix;). TRIzol® was purchased from Invitrogen (Thermo Fisher Scientific, Inc.). Antibodies against PI3K, P85, phospho-PI3K P85 (cat. no. Y607), insulin receptor β (IR), p-IR (cat. no. Y1185), PTEN, p-PTEN (cat. no. S380+T382+T383), glucose transporter 4 (cat. no. GLUT4), and IGF-1R were obtained from Abcam. Antibodies against Akt, p-Akt (ser 473), ERK, and p-ERK (1/2) (Thr202/Tyr204) were obtained from Cell Signaling Technology, Inc. Anti-GAPDH, anti-mouse IgG, and anti-rabbit IgG were obtained from Cell Signaling Technology, Inc. Other reagents used were of analytical grade.

### Animals and experimental design

Six-week old female db/db mice (18–20 g) were purchased from Model Animal Research Center of Nanjing University. All mice were housed individually with a 12-h light/dark cycle at 23 ± 2 °C and a humidity of 55 ± 10%, with free access to water and food. The mice were allowed to adapt to these conditions for 1 week prior to beginning the experimental procedures. The animal procedures were approved by the Animal Care and Use Committee on the Ethics of Animal Experiments of Taizhou University of Science and Technology. All related facilities and experimental procedures were performed according to the guidelines described by the Technical Standards for the Testing & Assessment of Health Food (2003).

### Cell culture

HUC-MSCs were provided by Jilin Tuohua Bioengineering Co. Ltd. (Siping). HUC-MSCs were immediately obtained from healthy mothers during routine term elective cesarean section births. Fully informed consent was obtained several weeks prior to delivery. Ethics approval was obtained from the Ethics Committee of the Siping Center Hospital (approval no. SC-2017-010). HUC-MSCs were isolated and propagated, as previously described [18].

### HUC-MSC treatment study

The db/db mice were divided into four groups: db/m (*n* = 10), db/m+HUC-MSCs (intraperitoneal injection, IP; *n* = 10), db/m+HUC-MSCs (tail vain injection, IV; *n* = 10), and db/m+HUC-MSCs (skeletal muscle injection, IM; *n* = 10); db/db (*n* = 15), db/db+HUC-MSCs (intraperitoneal injection, IP; *n* = 15), db/db+HUC-MSCs (tail vain injection), and db/db+HUC-MSCs (skeletal muscle injection). A total of 1 × 10^7^ HUC-MSCs (passage 4) were resuspended in 0.7 ml saline and administered by IP/IV; or 1 × 10^8^ (passage 4) in 2 ml saline were administered by local injection IM for the db/m+HUC-MSCs and db/db+HUC-MSCs groups. The other groups were given an equivalent volume of saline as the db/m and db/db groups, and both groups were fed a normal chow diet. To determine the effect of HUC-MSCs, survival rate, weight, blood glucose, ALT, AST, Cre, BUN, γ-GT, and the levels of serum insulin were measured at the mentioned times.

### Bioluminescence imaging

HUC-MSCs were infected with luciferase-lentivirus (CMV-Luciferase-PGK-Puro, cat. No. GM-0220PC, Shanghai Genomeditech Co., Ltd., Shanghai, China) to establish HUC-MSCs-luc cell stably expressing luciferase. The HUC-MSCs-luc skeletal muscle transplantation db/db mice were anesthetized using isoflurane and injected intraperitoneally with 100 μl of 15 mg/ml d-luciferin in Sodium salt D solution (Shanghai Yeasen Biotechnology Co., Ltd., Shanghai, China). Subsequently, the mice were imaged using an in vivo imaging system (FOBI; Berthold Technologies). In vivo imaging was performed prior to sacrifice.

### Calculation of IR index

IR calculated as follows: HOMA IR = serum insulin mmol/l × blood glucose mmol/l/22.5 [19].

### Histological examination of the skeletal muscle

A portion of the extracted skeletal muscle was immediately fixed in PBS mixed with 4% paraformaldehyde and embedded in paraffin. The sections (4 μm in thickness) were stained with hematoxylin and eosin (H&E). Histological analysis was performed using a light microscope (DM4000B photomicroscope; Leica Microsystems, Inc.).

### Immunofluorescence (IF) studies

Skeletal muscle sections were subjected to signal-direct IF staining of human DNA (1:10; ANA), followed by incubation with Alexa Flour 488-conjugated secondary antibodies (OriGene Technologies, Inc.). Nuclei were counterstained with Hoechst (Invitrogen; Thermo Fisher Scientific, Inc.). All sections were scanned, and images were acquired using a laser scanning confocal microscope (FV1000; Olympus Corporation).

### Immunohistochemical (IHC) staining

After de-paraffinization, the sections were incubated with a 3% H_2_O_2_ solution to block endogenous peroxidases. Antigen retrieval was performed using 0.1 M sodium citrate (pH 6.0) for 60 min. Sections were incubated with anti-PI3K, anti-GLUT4, anti-p-IR, anti-p-PTEN (1:100; Abcam), or anti-p-ERK (1:100; CST) antibodies overnight at 4 °C, and a horseradish peroxidase-conjugated secondary antibody and diaminobenzidine substrate were added sequentially. Following hematoxylin counterstaining and dehydration, the sections were mounted and observed using a Leica M4000B photomicroscope (Leica Microsystems, Inc.).

### ELISA

The concentrations of TNF-α, IL-6 (all from Abcam) and serum insulin (EMD Millipore) were determined using specific ELISA kits.

### Reverse transcription-quantitative (RT-q)PCR

Gene expression of c-fos, c-myc, JNK, GSK-3β, FOXO1, GLUT4, PTEN, and IRS-1in skeletal muscle section was analyzed using RT-qPCR. TRIzol® reagent was used to extract RNA, and cDNA was synthesized using a reverse transcription kit (Takara Bio, Inc.). qPCR containing was performed using a SYBR Premix EX Taq™ cDNA with specific gene primers, and genes were amplified using a 7300PULAS system (Applied Biosystems; Thermo Fisher Scientific, Inc.). The relative expression of each gene was determined and normalized to the expression of 18S and calculated using the 2^−ΔΔC^q method. The sequences of the primers used are as follows: 18S-forward 5′-ACTCAACACGGGAAACCTCAC-3′, and reverse, 5′-TCGCTCCACCAACTAAGAACG-3′; c-fos-forward, 5′-GGAATTAACCTGGTGCTGGATTG-3′, and reverse, 5′-GAACATTGACGCTGAAGGACTAC-3′; c-myc-forward, 5′-CTATCACCAGCAACAGCAGAG-3′, and reverse, 5′-ACATAGGATGGAGAGCAGAGC-3′; JNK-forward, 5′-ACATAGGATGGAGAGCAGAGC-3′, and reverse, 5′-CATTGACAGACGGCGAAGAC-3′; GSK-3β-forward, 5′-CACCGCTCCTTCCTTCCTTC-3′, and reverse, 5′-GACTCCTCTTCCTAACCACCTG-3′; FoxO1-forward, 5′-CAGCCTTGAGCAGCCTAATG-3′, and reverse, 5′-AGACTGGGAAACACCGATGG-3′; GLUT4-forward, 5′-ACGGATAGGGAGCAGAAACC-3′, and reverse, 5′-CAGCACAGGACACTCATCTTC-3′; PTEN-forward, 5′-AGAGATTGGCTGCTGTCCTG-3′, and reverse, 5′-TGGTTAAGTCATTGCTGCTGTG-3′; IRS-1-forward, 5′-AGCAGCAGTAGCAGCATCAG-3′, and reverse, 5′-TTACCGCCACCACTCTCAAC-3′; IGF-1-forward, 5′-TATGGAGATGGGAGGGTTTCAG-3′, and reverse, 5′-GTAGGCACAGCATTCGTTAGG-3′; mTOR-forward, 5′-GCAGCAACAGTGAGAGTGAAG-3′, and reverse, 5′-CAAGGAGATAGAACGGAAGAAGC-3′.

### Western blot analysis

Protein samples from skeletal muscle section were resolved using 8 or 10% SDS-PAGE and transferred to PVDF membranes (EMD Millipore). Membranes were blocked in 5% nonfat dry milk and subsequently incubated with primary antibodies against p-PI3K/PI3K, p-AKT/Akt, p-IR/IR, p-PTEN/PTEN and IGF-1R (1:500; Abcam), p-ERK/ERK1/2, and GAPDH (1:500; CST Biological Reagents Co., Ltd.) overnight. Horseradish peroxidase-conjugated anti-rabbit IgG or anti-mouse IgG (1:7000; CST Biological Reagents Co., Ltd.) were used as the secondary antibodies. PVDF membranes were developed using an Image-ProPlus system.

### Statistical analysis

Data are represented as the mean ± standard deviation and analyzed using a one-way ANOVA or a two-tailed unpaired Student’s *t* test. *P* values were adjusted for multiple comparisons using Bonferroni correction. Analyses were performed using GraphPad Prism version 7 (GraphPad Software, Inc.). *P* < 0.05 was considered to indicate a statistically significant difference.

## Results

### Therapeutic effects of transplanted HUC-MSCs in db/db mice

To evaluate the potential capacity of HUC-MSCs and the effect of administration route, FBG, weight, and survival rates were assessed. The HUC-MSCs were administered by three clinically relevant routes: IV, IP, and IM. These different transplantation routes resulted in different FBG levels, but did not have any effect on weight in the db/db mice. Although administration of cells using IV was the best method for controlling FBG, IV administration resulted in the lower survival rate of 71.9%. HUC-MSC transplantation by IM also reduced the FBG, and the survival rate was 100%. Thus, IM transplantation was deemed to be the safest and most effective method of clinical application of HUC-MSCs.

In addition, HUC-MSC transplantation did not result in injury to the liver and kidneys in mice. The levels of ALT, AST, γ-GT, Cre, and BUN in serum were measured 72 h after HUC-MSC transplantation (Table 1). The results found that HUC-MSC transplantation did not have any adverse effects on liver and kidney toxicity regardless of the administration routes.

**Table 1:**
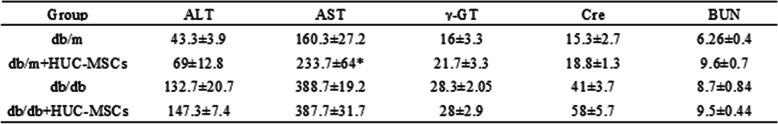
Effects of HUC-MSC transplantation on liver and kidney function in experimental mice

Data are presented as the mean ± standard deviation. db/m vs db/m+HUC-MSCs, *P < 0.05; **P < 0.01; db/db vs db/db+HUC-MSCs, ^**#**^*P* < 0.05; ^**##**^*P* < 0.01

*HUC-MSC* human umbilical cord-mesenchymal stem cells

### HUC-MSC treatment ameliorates IR in the skeletal muscle of db/db mice

**Fig. 2:**
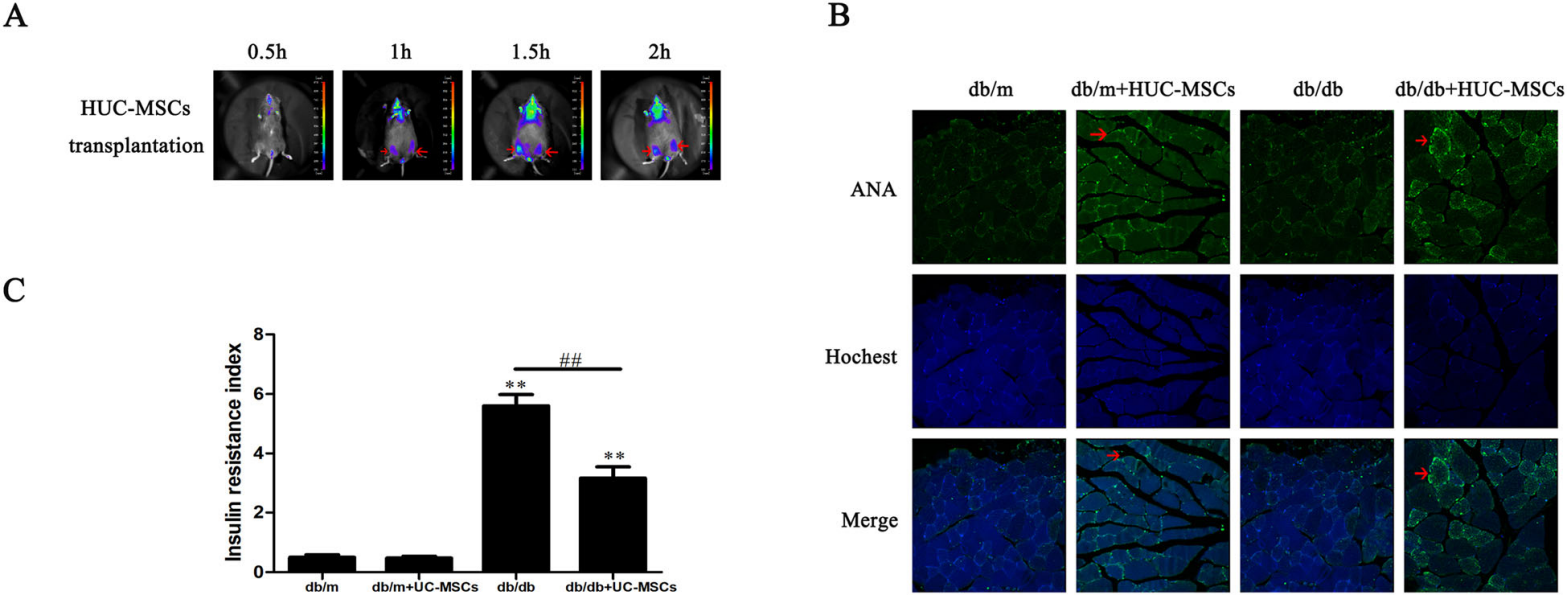
HUC-MSC transplantation ameliorates IR in the skeletal muscle of db/db mice. **a** Bioluminescence imaging detected the colonization of HUC-MSCs following IM transplantation, red arrows show colonization of HUC-MSCs. **b** Skeletal muscle tissue sections from each mouse were stained with ANA and assessed using immunofluorescence, ANA (green), nuclear (blue), red arrows show the positive of ANA. Magnification, × 400. **c** Insulin resistance index. The results are representative of 3 independent repeats. Data are presented as the mean ± standard deviation. **P < 0.01 vs. baseline levels in db/m. ^##^*P* < 0.01. HUC-MSC, human umbilical cord-mesenchymal stem cells; IR, insulin resistance

To facilitate longitudinal cell tracking in vivo imaging, HUC-MSCs were infected with luciferase-lentivirus (CMV-Luciferase-PGK-Puro) to establish HUC-MSCs-luc cell stably expressing luciferase. The results showed that HUC-MSCs stably colonized in the skeletal muscle following IM transplantation. Although fluorescent signals were detected in the bladder region 1.5 h after transplantation, these signals were absent after 2 h (Fig. 2a). Subsequently, the proliferation and differentiation of HUC-MSCs in skeletal muscle were assessed. The HUC-MSCs were labeled using an anti-nuclear antibody (ANA), and the expression of ANA was determined using IF. Consistent with Braid et al [20], ANA expression in skeletal muscles were significantly increased following IM implantation in the db/db+HUC-MSC mice compared with the db/db mice (Fig. 2b). Thus, IM implantation highlights the potential clinical benefits of the prolonged presence of MSCs at a homeostatic implant site using a minimally invasive delivery route suitable for numerous applications.

Subsequently, IR was calculated and the mean HOMA IR was significantly decreased in db/db+HUC-MSC mice compared with db/db mice (*P* < 0.01). However, HUC-MSC implantation did not significantly alter the HOMA IR in the db/m mice (Fig. 2c).

### HUC-MSC treatment alleviates inflammation of the skeletal muscle in db/db mice

**Fig. 3:**
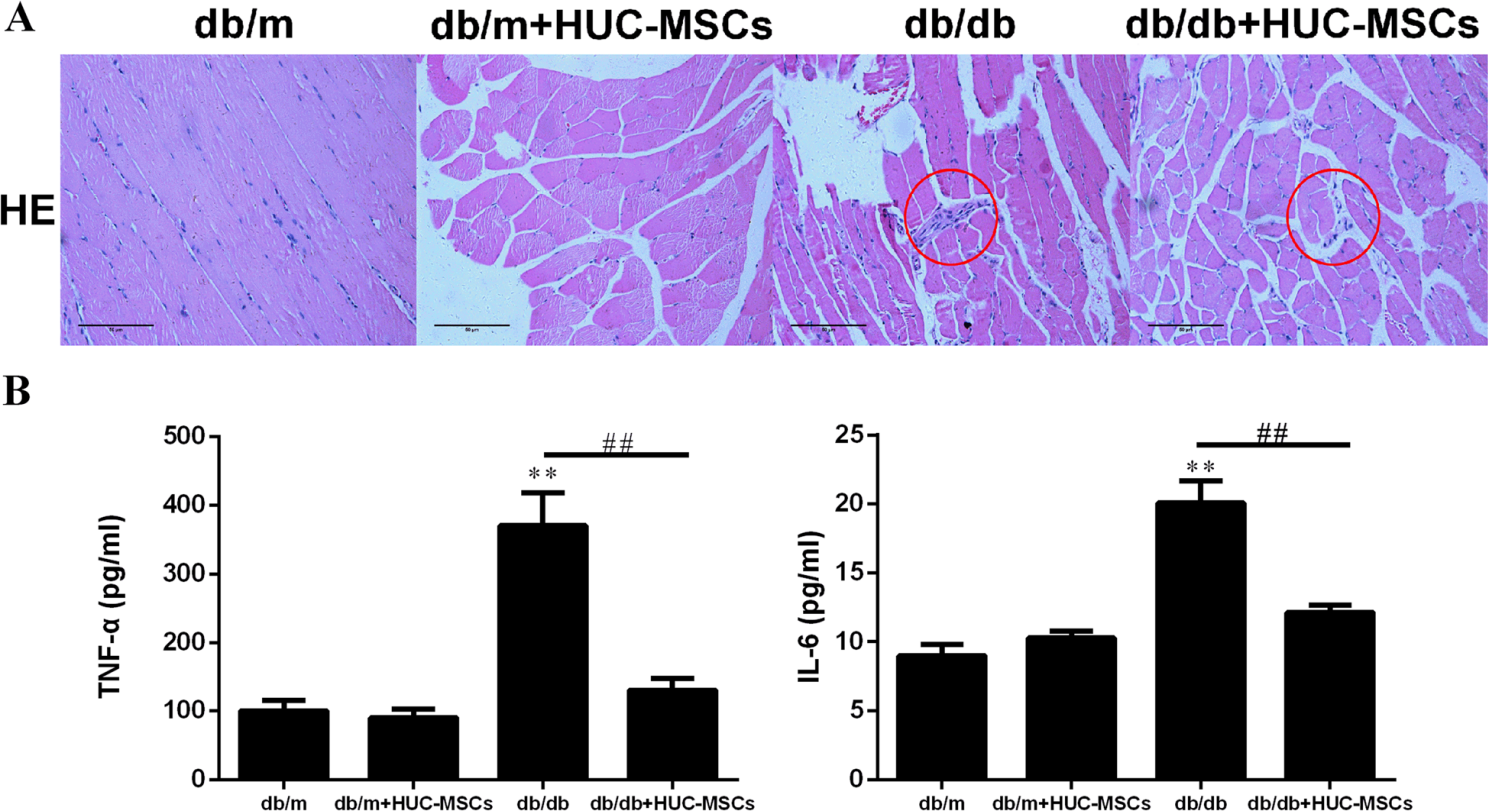
HUC-MSC treatment alleviates inflammation of the skeletal muscle in db/db mice. **a** Skeletal muscle tissue sections from each rat were stained with hematoxylin and eosin, red circle show the inflammation of the skeletal muscle. **b** TNF-αand IL-6 levels in the serum of each group. The results are representative of 3 independent experiments. Data are presented as mean ± standard deviation. Data are presented as the mean ± standard deviation. **P < 0.01 vs. baseline levels in db/m. ^##^*P* < 0.01. HUC-MSC, human umbilical cord-mesenchymal stem cells; IR, insulin resistance

In the present study, inflammatory cell infiltration was significantly higher in the db/db compared with the db/db+HUC-MSC mice (Fig. 3a). The secretion of IL-6 and TNF-α are shown in Fig. 3b. There were no significant differences in the secretion of any of the factors between db/m and db/m+HUC-MSC mice. However, the expression levels of IL-6 and TNF-α were significantly lower in db/db mice with HUC-MSC transplantation compared with the untransplanted mice (*P* < 0.01; Fig. 3b).

### HUC-MSC treatment ameliorates IR through regulating the activation of PI3K/Akt signaling in the skeletal muscle of db/db mice

**Fig. 4:**
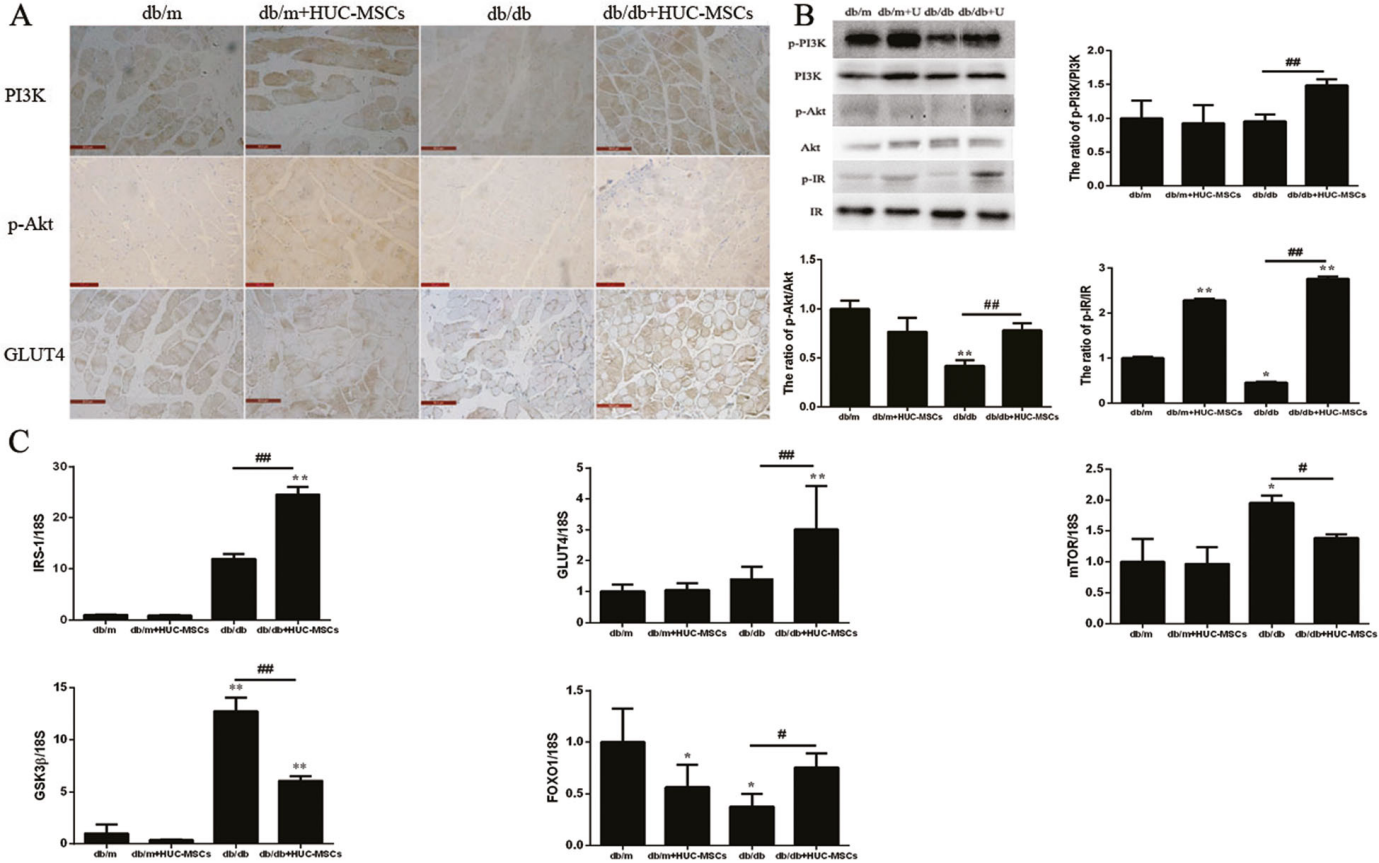
HUC-MSC treatment ameliorates IR through regulating the activation of PI3K/Akt signaling in the skeletal muscle of db/db mice. **a** The intensity of immunohistochemical staining for PI3K, p-Akt, and GLUT4 were evaluated based on the optical density by fixed skeletal muscle tissues. Representative images tissues from mice in each group are presented. Magnification, × 400. **b** Protein expression levels of PI3K, p-PI3K, Akt, p-Akt, IR, and p-IR in skeletal muscle tissues. **c** mRNA expression levels of IRS, GLUT4, GSK3β, FoxO1, and mTOR. *P < 0.05 **P < 0.01 vs. the baseline levels (db/m). Data are presented as the mean ± standard deviation. ^#^*P* < 0.05, ^##^*P* < 0.01 between db/db and db/db+HUC-MSCs. The results are representative of 3 independent repeats. HUC-MSC, human umbilical cord-mesenchymal stem cells; IR, insulin receptor

To further investigate the effect of HUC-MSC implantation on the PI3K/Akt signaling pathway, the expression levels of members of the PI3K/Akt signaling pathway in skeletal muscle tissue were assessed using IHC, western blotting and RT-qPCR. The IHC results showed that the expression of PI3K, p-Akt, and GLUT4 were increased in the db/db+HUC-MSC mice compared with the db/db mice (Fig. 4a). The expression of p-PI3K/PI3K, p-Akt/Akt, and p-IR/IR in skeletal muscle tissue were assessed using western blotting, and the ratio of p-PI3K/PI3K, p-Akt/Akt, and p-IR/IR expression were increased in the db/db+HUC-MSC mice compared with the db/db mice (*P* < 0.05 and *P* < 0.01; Fig. 4b). The mRNA expression levels of IRS-1, GLUT4, and FoxO1 were noticeably increased in the db/db mice transplanted with HUC-MSCs, and the mRNA expression levels of mTOR and GSK-3β were significantly decreased in the db/db mice transplanted with HUC-MSCs compared with db/db mice (*P* < 0.05 and *P* < 0.01; Fig. 4c).

### HUC-MSC treatment ameliorates IR through regulating the activation of ERK/MAPK signaling in the skeletal muscle of db/db mice

**Fig. 5:**
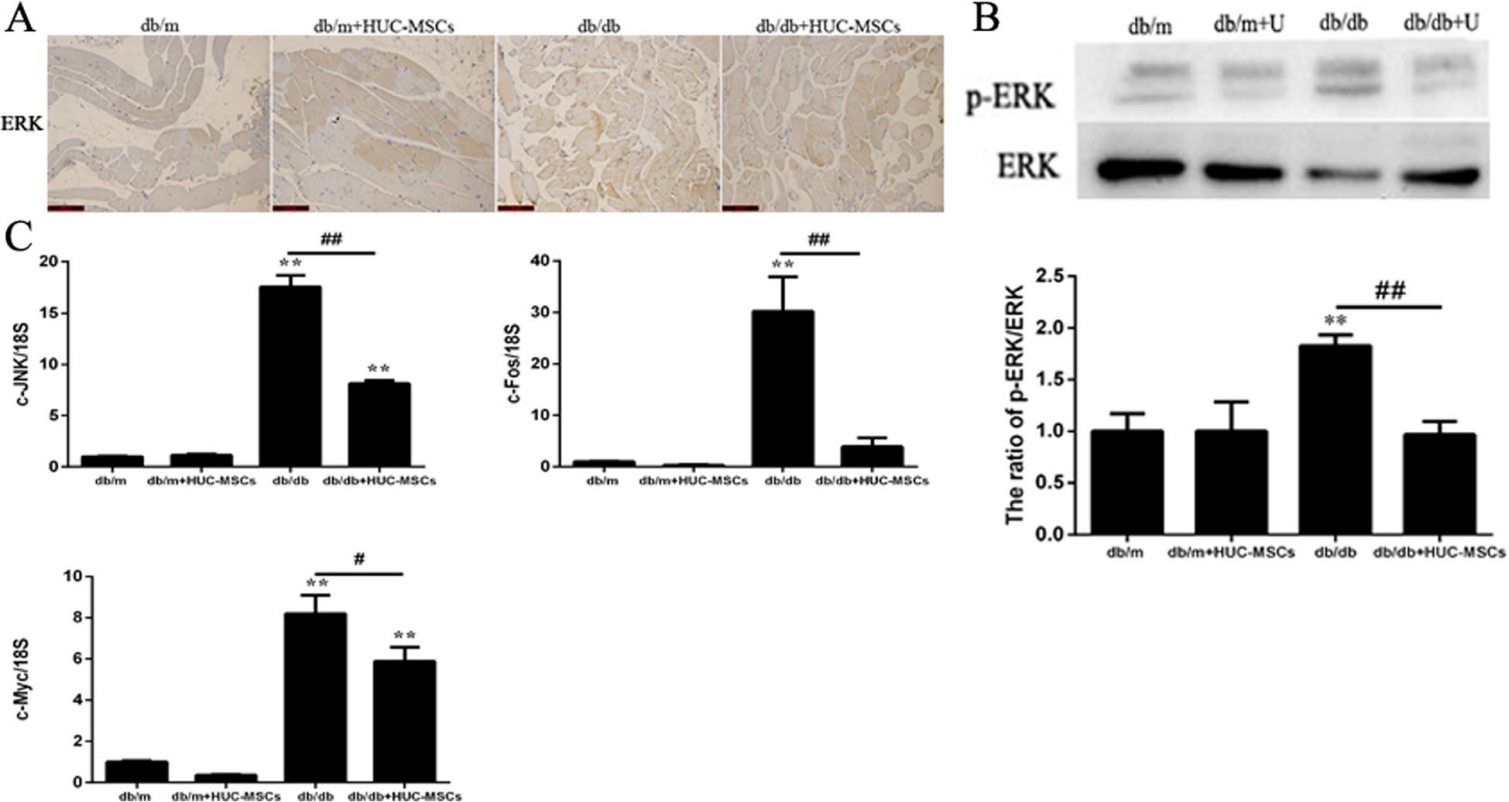
HUC-MSC treatment ameliorates IR through regulating the activation of ERK/MAPK signaling in the skeletal muscle of db/db mice. **a** Fixed skeletal muscle tissues were stained with ERK, and the intensity of immunohistochemical staining for ERK was evaluated by optical density. Representative images tissues from mice in each group. Magnification, × 400. **b** Skeletal muscle tissues were subjected to western blotting using specific antibodies against ERK and p-ERK. **c** mRNA expression levels of JNK, c-fos, and c-myc. Results are representative of 3 independent repeats. Data are presented as the mean ± standard deviation. *P < 0.05, **P < 0.01 vs. the baseline levels (db/m); ^#^*P* < 0.05 and ^##^*P* < 0.01

To investigate the effects of HUC-MSC implantation on the ERK/MAPK signaling pathway, the expression levels of members of the ERK/MAPK signaling pathway were determined in skeletal muscle tissues using IHC, western blotting, and RT-qPCR. IHC results showed that the expression of ERK was decreased in db/db+HUC-MSCs mice compared with db/db mice (Fig. 5a). The expression of p-ERK/ERK in skeletal muscle tissues was measured using western blotting, and the ratio of p-ERK/ERK expression was also decreased in the db/db+HUC-MSCs mice compared with db/db mice (*P* < 0.01; Fig. 5b), whereas the mRNA expression levels of JNK, c-fos, and c-myc were significantly decreased in the db/db mice transplanted with HUC-MSCs compared with the db/db mice (*P* < 0.05,*P* < 0.01; Fig. 5c).

### HUC-MSC treatment regulates the balance between PI3K/Akt and Erk/MAPK signaling by increasing PTEN transcription and expression in the skeletal muscle of db/db mice

**Fig. 6:**
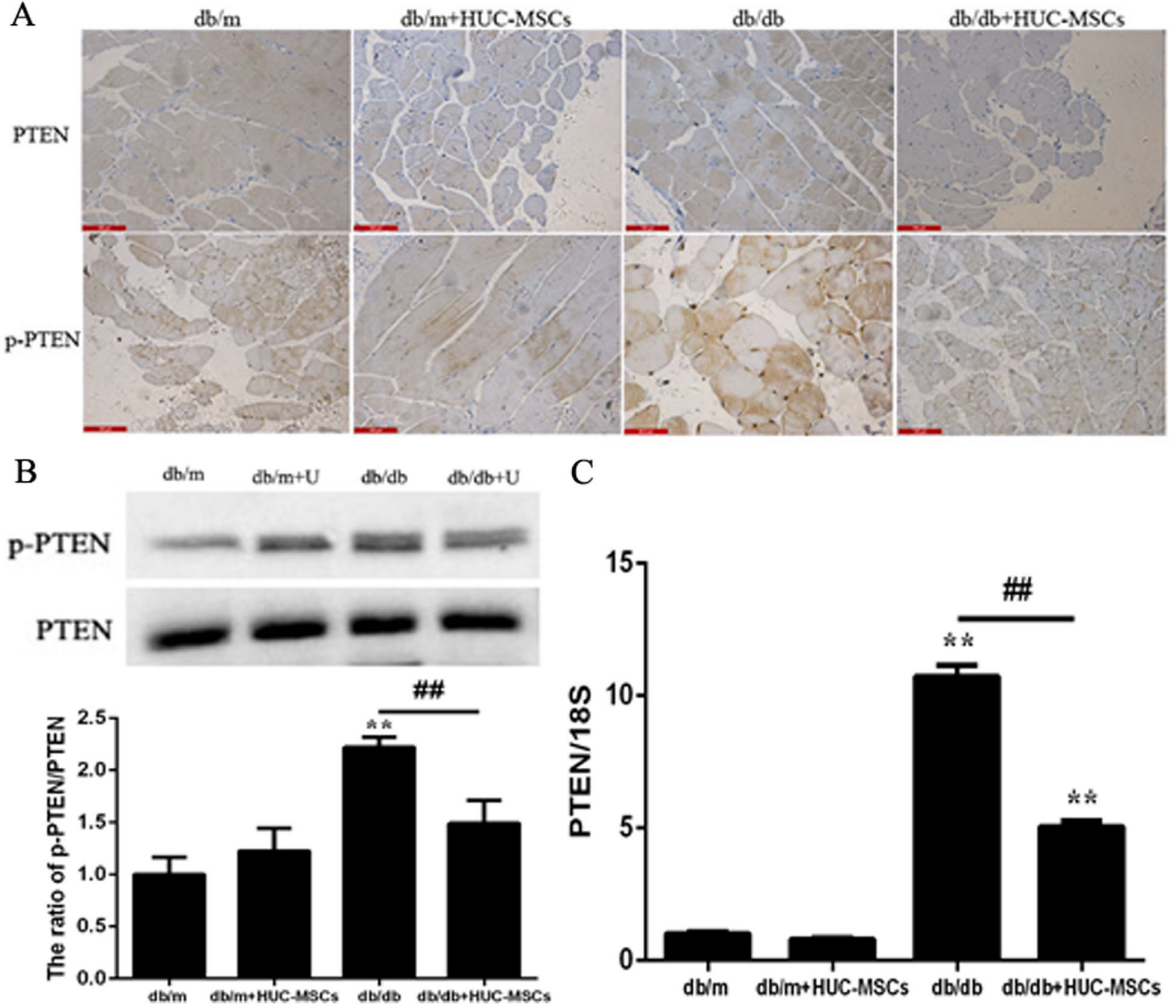
HUC-MSC treatment regulates the balance between PI3K/Akt and Erk/MAPK signaling by increasing PTEN expression in the skeletal muscle of db/db mice. **a** Fixed skeletal muscle tissues were stained with PTEN and p-PTEN antibodies, and the intensity of immunohistochemical staining was evaluated. Representative images of tissues from each group. Magnification, × 400. **b** PTEN and p-PTEN protein expression levels in skeletal muscle tissues. **c** mRNA expression levels of PTEN in skeletal muscle tissues. Results are representative of 3 independent repeats. Data are presented as the mean ± standard deviation. *P < 0.05, **P < 0.01 vs. the baseline levels (db/m). ^#^*P* < 0.05, ^*##*^*P* < 0.01

To further investigate the effect of PTEN on PI3K/Akt and Erk/MAPK signaling in db/db mice with HUC-MSC transplantation, the effect of PTEN in skeletal muscle tissues was determined. The results showed that the protein expression levels of PTEN and p-PTEN were decreased in db/db+HUC-MSC mice compared with db/db mice (Fig. 6a, b; *P* < 0.01). In addition, the mRNA expression levels of PTEN exhibited similar changes as the changes in the protein expression levels (Fig. 6c).

### HUC-MSC treatment ameliorates IR, but this is not associated with the IGF-1/IGF-1Rpathway

**Fig. 7:**
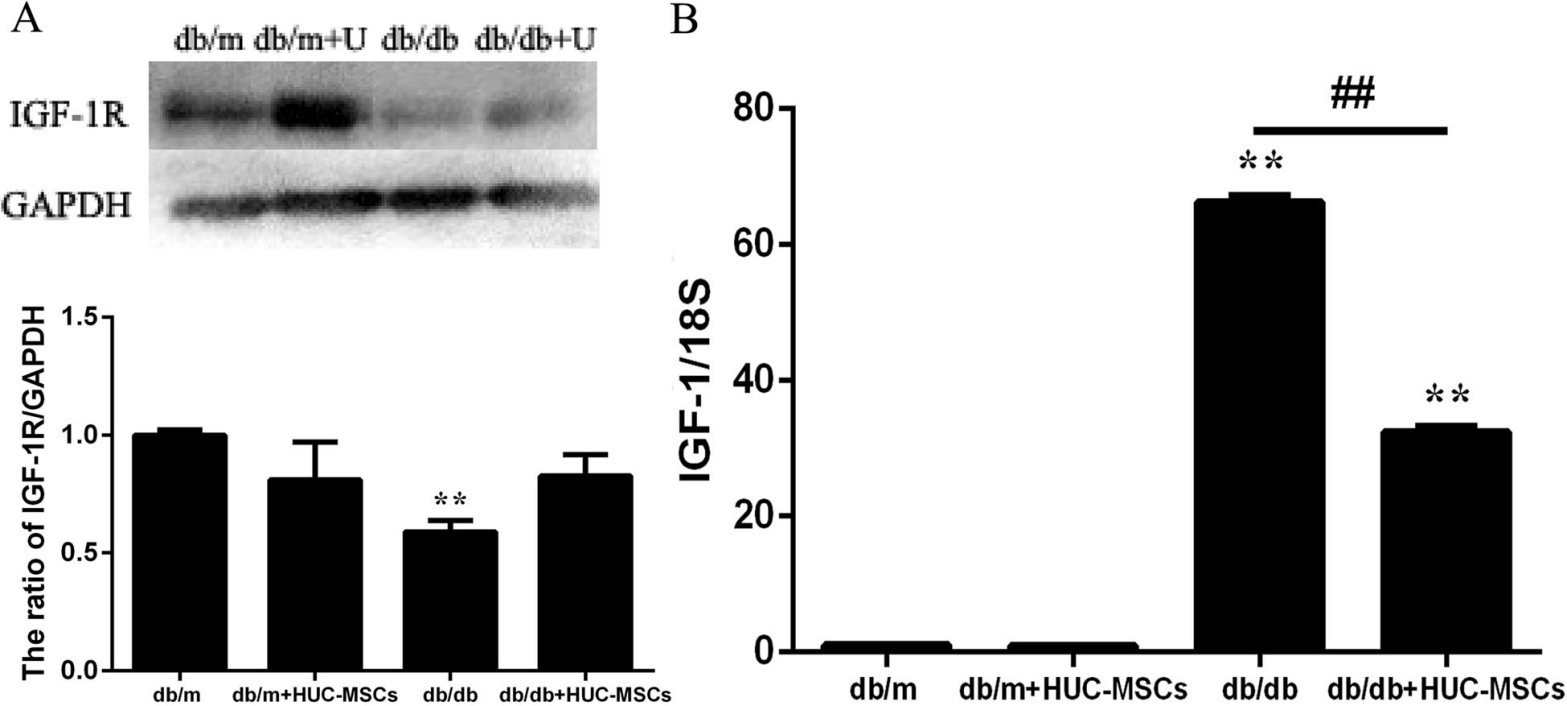
HUC-MSC treatment ameliorates IR, and this does not involve the IGF-1/IGF-1R pathway. **a** Protein and **b** mRNA expression levels of IGF-1R in skeletal muscle tissues. Results are representative of 3 independent repeats. Data are presented as the mean ± standard deviation. *P < 0.05, **P < 0.01 vs. the baseline levels (db/m). ^#^*P* < 0.05, ^##^*P* < 0.01

To determine whether HUC-MSC transplantation improved insulin sensitivity was associated with the IGF-1/IGF-1R pathway, the expression of IGF-1 and IGF-1R in skeletal muscle tissue of db/db+HUC-MSC mice was assessed. Only the expression of IGF-1 mRNA was reduced by HUC-MSC implantation compared with db/db mice (*P* < 0.01; Fig. 7b). However, HUC-MSC transplantation did not result in changes to the protein expression levels of IGF-1R in db/db+HUC-MSC mice (Fig. 7a).

## Discussion

IR is a central defect in metabolic syndromes such as T2DM and serves a critical role in the progression from pre-T2DM to T2DM. Despite the development of multiple drugs to ameliorate IR, the limitations of the accompanying side effects are still notable and should not be ignored. Therefore, development of a more effective method to improve IR is required. Recently, there has been a focus on the link between HUC-MSC transplantation and IR [15]. The present study investigated the anti-IR effects and potential mechanisms of HUC-MSC transplantation in db/db mice in vivo. The results showed that IM HUC-MSC transplantation was safer compared with IV and IP administration, and the survival rate of IM transplantation reached 100%. HUC-MSCs stabilized localization and differentiation in the skeletal muscle and significantly ameliorated IR. Potential regulatory mechanisms underlying the effects of HUC-MSCs were associated with downregulation of inflammation, and PTEN-mediated regulation of the balance between PI3K/Akt and ERK/MAPK signaling pathways, and were not associated with the IGF-1/IGF-1R pathway. This suggests that HUC-MSCs may be a potential novel therapeutic agent for the clinical prevention of IR and associated diseases.

MSCs, as a source of seed cells, have garnered increasing attention in the field of stem cell therapy. At present, a large number of studies have discussed the clinical efficacy of stem cell therapy [21–23]; however, the safety and effectiveness of the optimal route for HUC-MSC transplantation have not been extensively considered. The safety of MSC administration has been reported in numerous clinical trials [24–30]. At present, IV infusion is the standard practice for delivering cell therapies. To be mentioned, researchers found that MSCs can be trapped largely in the capillaries of the lungs by IV transfusion, resulting in death after a few days [15, 29, 31–35]. This phenomenon prompted us that MSC transfusion by IV limited the potential therapeutic benefit in both clinical trials and animal studies [27, 36, 37]. However, engineered MSC transplantation by IM can live last for more than 100 days, and they perform their function by secreting a functional antibody into circulation [38]. Thus, in the present study, three routes of HUC-MSC transplantation in db/db mice were compared. Although administration of cells using IV was the best method for controlling FBG, the survival rate of db/db mice was 100% with IM transplantation compared with 71.4% for IV and 85.7% for IP. Additionally, HUC-MSCs stabilized localization, proliferation, and differentiation in the skeletal muscle. IM implantation presents a useful alternative to achieve clinical benefits from prolonged MSC presence at a homeostatic implant site and is a minimally invasive delivery route suitable for several applications [18]. Reaven et al. suggested that IR, manifesting as hyperinsulinemia, is the driving factor for the development of dyslipidemia, elevated blood pressure, and altered glucose metabolism [39]. In this experiment, HUC-MSCs significantly ameliorated IR (HOMA-IR) via IM injection in db/db mice, inducing obviously the downregulation of FBG. Thus, IM administration was shown to be more preferable than IV or IP infusion for treatment of IR and other related diseases. However, optimized thawing protocols that restore the full biological potential of cryopreserved MSC therapies prior to implantation must be developed.

Low-grade chronic inflammation can be found in adipose tissue, skeletal muscle, liver, pancreas and the hypothalamus, correlated with nutrient overload [40]. However, low-grade systemic chronic inflammation also correlated with the development of IR, which has a central role in the pathogenesis of T2DM [41, 42]. In addition, pro-inflammatory cytokines took part in the activation or inhibition process of insulin signaling at the molecular and cellular level [43, 44]. Research from Hotamisligil et al. [45] found that the development of IR was associated with inflammation, in which level of TNF-α raised in a T2DM rodent model. Obesity elevated levels of C reactive protein and IL-1β in the blood, just increasing the development risk of T2DM [45, 46]. Thus, the development of IR and T2DM can be induced by chronic inflammation, which has been recognized as a critical inducer. Consistently, in the animal experiments of the present study, HUC-MSC transplantation significantly decreased the inflammatory response in skeletal muscle tissue of db/db mice and decreased the levels of TNF-α and IL-6 production in the peripheral blood compared with those in the model group. Thus, these findings suggest that HUC-MSC transplantation can decrease a low-degrade inflammatory response, and this may underlie the amelioration of IR.

In addition, IR induced the development of obesity and T2DM, which is correlated with damage of PI3K/AKT pathway in various tissues of the body, following IR aggravates the PI3K/AKT pathway into a vicious circle [47]. Upon insulin stimulation, two signaling pathways are activated. Firstly, insulin binding the insulin receptor leads to activation of the PI3K/Akt pathway, which regulates glucose metabolism, including glucose uptake and glycogenesis [48–51]. Secondly, insulin also induces activation of the MAP kinases (Erk 1 and 2) and SAP kinases (JNK and p38MAPK), which downregulates the activation of insulin signaling, correlated with IR [52–54]. In the present study, HUC-MSC transplantation significantly induced activation of PI3K/Akt signaling, thus increasing the expression of p-PI3K, p-Akt, GLUT4, and p-IR protein compared with db/db mice. Additionally, the expression of IRS, GLUT4, and FoxO1 mRNA were increased in db/db mice transplanted with HUC-MSCs. However, the expression levels of GSK3β and mTOR were decreased in db/db mice transplanted with HUC-MSCs compared with the db/db mice (Fig. 4c). IRS proteins, which are crucial regulators of the insulin and IGF signaling pathways, are important in the linkage of membrane receptors to intracellular substrates and can activate different intracellular signaling pathways, including PI3K [43, 55]. Moreover, studies have shown that disruption of GLUT4 expression in adipose tissues or muscles results in global IR [56]. Importantly, in skeletal muscle cells, the quantity of GLUT4 is largely controlled at the level of transcription [57]. Furthermore, accumulating evidence suggests that FoxO1 is a key transcription factor responsible for regulation of insulin signaling, and knockdown of FoxO1 improved insulin sensitivity [58]. In the present study, HUC-MSC transplantation increased the expression of FoxO1 mRNA compared with db/db mice, although the expression levels were lower compared with the db/m mice. However, studies have reported that mTORC2 phosphorylates and activates Akt, and mTORC1 levels are decreased by suppression of tuberous sclerosis protein 2 following the activation of Akt. mTORC2 suppress FoxO1 forkhead transcription factor promotes gluconeogenesis, mediating the effects of insulin on the suppression of hepatic glucose production [59]. In contrast to these previous studies, the mRNA expression levels of total mTOR and GSK3β mRNA were decreased in the db/db+HUC-MSCs mice; the primary reason for this inconsistency may be that total mTOR mRNA levels were detected, not mTORC1 or mTORC2 mRNA individually. In addition, activation of Akt serves an important role in fundamental cellular functions, such as cell proliferation and survival by phosphorylating its downstream substrates, including GSK3β, and thereby, inactivating them [60]. Thus, the results of the present study showed that HUC-MSC transplantation improved IR, and this was associated with a decrease in the activity of the PI3K/Akt signaling pathway.

Numerous studies found that MAPK-dependent signal transduction is crucial for an array of metabolic events and inappropriate MAPK signaling is associated with the development of T2DM and metabolic syndrome [43]. HUC-MSC transplantation significantly decreased activation of the ERK/MAPK signaling pathway, thus decreasing the expression of p-ERK compared with db/db mice. Additionally, the mRNA expression levels of JNK, c-fos, and c-myc mRNA were decreased in the db/db mice transplanted with HUC-MSCs. ERK activity has a critical role in the development of IR, which can be influenced by diabetogenic factors; hence, downregulating ERK activity obtains a potential therapeutic advantage to treat IR [61]. Moreover, researchers also found the important role of JNK in adiposity, metabolic inflammation, and IR [43]. JNK can regulate phosphorylation of IRS, promote metabolic inflammation, and inhibit the pituitary thyroid axis, PPARα activity, and fibroblast growth factor 21 expressions. Thus, knock-down JNK can improve insulin signaling and glucose tolerance, which is a potential therapeutic approach to IR [62]. In the present study, it was shown that HUC-MSC transplantation decreased the activation of ERK and JNK expression. Therefore, HUC-MSC transplantation may be a therapeutic approach to improve IR.

Accumulating evidence has suggested that the PI3K/Akt and ERK/MAPK pathways are activated due to a loss of phosphatase and PTEN function [63], and PTEN is a negative regulator of the PI3K signaling pathway [64, 65]. To evaluate the potential capacity of PTEN in regulating the balance between PI3K/Akt and ERK/MAPK pathway in HUC-MSCs treated db/db mice, the expression of PTEN was assessed using IHC, western blotting, and RT-qPCR. The expression levels of PTEN and p-PTEN protein were decreased in the db/db+HUC-MSC mice compared with db/db mice. In addition, the mRNA expression levels of PTEN were also decreased. Consistently, upregulation of PTEN expression inhibited the activation of the PI3K/Akt signaling pathway, increased the activation of the ERK/MAPK signaling pathway, and thus increased the incidence of IR. The balance between these two signaling pathways is considered a key regulator of insulin sensitivity [66, 67]. Therefore, identifying the key regulator for the coordination between these two signaling pathways may not only shed light on the mechanisms underlying IR, but also provide novel strategies for treatment of diabetes. PTEN just is a potential regulator of the coordination between PI3K/Akt and MAPK signaling pathways, providing novel insights into the development of potential therapeutics for IR in diabetic patients in the future.

Nevertheless, IGF-1R serves a significant role in insulin signaling. IGF-1 and IGF-1R are known to maintain insulin sensitivity in different tissues [43]. IR seems to be essential in the pathophysiological process, and for this reason, based on the information available, we propose IGF-1 as a key hormone in the pathophysiology of metabolic syndrome due to its implications in the metabolism of carbohydrates and lipids [68].IGF-1 is a homologue molecule that acts predominantly as an anabolic hormone involved in cell growth, differentiation, and migration [69]. IGF-1 exerts its biological functions through activation of IGF-1R-mediated downstream signaling pathways. IGF-1 exhibits ~ 50% amino acid sequence homology with insulin and elicits a similar hypoglycemic response [70]. In the present study, the expression of IGF-1 mRNA was decreased by HUC-MSC implantation compared with the db/db mice. However, HUC-MSC transplantation did not alter the expression of IGF-1R protein in the db/db+HUC-MSCs mice. These results suggest that HUC-MSC transplantation-mediated improvement of IR in the skeletal muscle tissues of db/db mice was not associated with regulation of the IGF-1/IGF-1R signaling pathway.

## Conclusions

Together, these results demonstrate that IM HUC-MSC transplantation is the safer than IV or IP administration, and IM administration resulted in 100% survival rate of mice. HUC-MSCs can stabilize localization and differentiation in skeletal muscle tissue and significantly ameliorate IR, and this was mediated by PTEN-mediated regulation of the balance between PI3K-Akt and Erk/MAPK signaling pathways. Furthermore, HUC-MSC implantation also downregulated the expression of inflammatory markers in the skeletal muscle tissue. Therefore, HUC-MSC transplantation may be a potential therapeutic direction for preventing IR and increasing insulin sensitivity.

## Data Availability

The datasets supporting the conclusions of this article are included within the article.

## Abbreviations

HUC-MSCs: Human umbilical cord-derived mesenchymal stem cells
T2DM: Type 2 diabetes mellitus
IR: Insulin resistance
IV: Tail vein injection
IP: Intraperitoneal injection
IM: Skeletal muscle injection
GLUT4: Glucose transporter 4
IR: Insulin receptor
H&E: Hematoxylin and eosin
IF: Immunofluorescence
IHC: Immunohistochemical
RT-qPCR: Reverse transcription-quantitative PCR
ANA: Anti-nuclear antibody
IL-6: Interleukin-6
TNF-α: Tumor necrosis factor-α
IRS-1: Insulin receptor substrate-1
IGF-1: Insulin-like growth factor-1
